# Revealing the Role of Interfacial Charge Transfer in Mechanoluminescence

**DOI:** 10.3390/nano15090656

**Published:** 2025-04-26

**Authors:** Xinyi Huo, Shaoxin Li, Bing Sun, Zhonglin Wang, Di Wei

**Affiliations:** 1 School of Science, China University of Geosciences, Beijing 100083, China; huoxy@email.cugb.edu.cn (X.H.); sunbing@cugb.edu.cn (B.S.); 2 Beijing Institute of Nanoenergy and Nanosystems, Chinese Academy of Sciences, Beijing 101400, China; lishaoxin@binn.cas.cn; 3 School of Nanoscience and Engineering, University of Chinese Academy of Sciences, Beijing 100049, China

**Keywords:** mechanical luminescence, contact-electro-luminescence, luminescence, charge transfer, contact electrification

## Abstract

Mechanoluminescence (ML) involves light emission induced by mechanical stress, categorized into triboluminescence (TL), piezoluminescence (PL), sonoluminescence (SL), and triboelectrification-induced electroluminescence (TIEL). The most common is TL, in which crystal fracture generates opposing charges that excite surrounding molecules. In PL, applied pressure induces light emission via charge recombination. SL occurs in gas-saturated liquids under sudden pressure changes. TIEL has gained increasing attention as it operates without the need for asymmetric crystal structures or strain fields. However, conventional ML faces practical limitations due to its dependence on complex structures or strain fields. In contrast, contact-electro-luminescence (CEL) has emerged as a promising alternative, enabling luminol luminescence via charge transfer and reactive oxygen species generation through contact electrification (CE) between inert dielectrics and water. CEL provides a simpler and more versatile approach than traditional ML techniques, underscoring the pivotal role of charge-transfer processes. This perspective highlights the potential of CEL in expanding ML applications across sensing, energy conversion, and environmental monitoring.

## 1. Introduction

**Figure 1:**
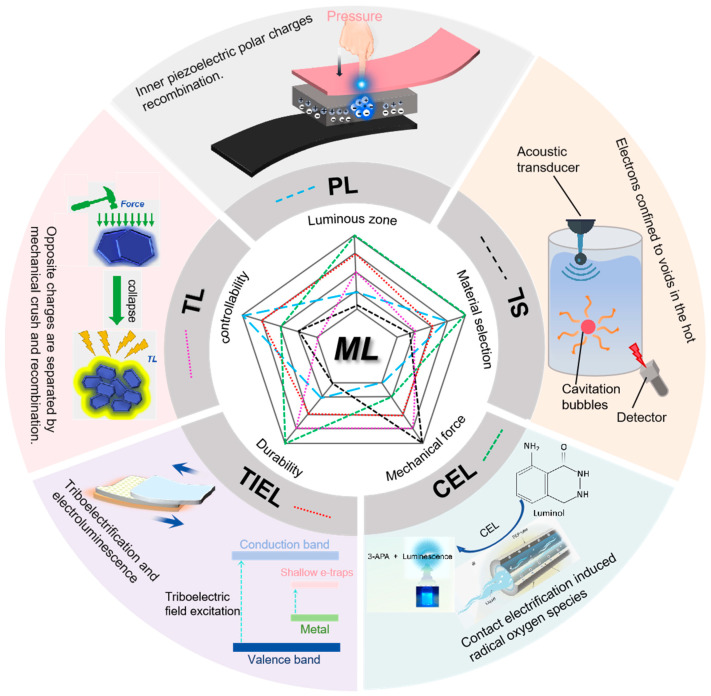
A schematic diagram of various ML methods with their respective characteristics.

Mechanoluminescence (ML) occurs when certain materials emit light in response to mechanical stimuli such as bending, stretching, or compression [1,2]. As a compelling physical phenomenon, the mechano-to-light conversion in ML holds significant research value and promises broad applications in biological sensing, electronic sensors [3], etc. First documented in 1605 by Francis Bacon, who observed luminescence when scraping hard candy with a knife [4], ML has been studied for centuries. Over time, it has been identified in nearly one-third of organic molecular solids [5] and half of inorganic salts, spanning conductors, semiconductors, and insulators. In 1999, Zhang et al. introduced the concept of trap-controlled materials, exemplified by ZnS:Mn^2+^ and SrAlO_4_:Eu^2+^. These materials contain luminescent ions embedded within an inorganic host matrix, where the presence of structural defects or intentional dopants creates electron or hole traps [6]. Upon mechanical stimulation, the trapped charges are released, triggering light emission [7]. Most trap-controlled materials are derived from aluminate, sulfide, silicate, titanate, and phosphate matrices, while fewer examples involve gallate, oxide, nitroxide, stannate, oxysulfide, or germanium silicate hosts. They are characterized by intense ML emissions across the ultraviolet–near-infrared spectrum, demonstrating multi-scale sensitivity to mechanical stress [8]. Today, ML has found widespread applications in fluorescent switches [9], mechanical sensors [10], protective coatings, optoelectronic devices [11], and even data storage, reinforcing its growing significance in both scientific research and industrial practice [12]. Understanding the fundamental physics governing mechano-to-light conversion in ML materials has become crucial for material innovation and practical applications in the 21st century [13]. However, a unified explanation remains elusive due to the intricate interplay between mechanical stimuli and the multiple light-emission pathways involved in ML. Mechanistic interpretations have been phenomenologically or empirically attributed to effects such as ultrasonication-induced cavitation, piezoelectricity, and triboelectrification. Based on the mode of luminescence induction, ML can be classified into piezoluminescence (PL), sonoluminescence (SL), triboluminescence (TL), and triboelectrification-induced electroluminescence (TIEL), among others (Figure 1) [14]. While these distinct ML processes have attracted considerable attention and have demonstrated promising applications, their underlying charge-transfer mechanisms remain insufficiently understood. Notably, ML is invariably associated with dynamic interfacial evolution between materials under mechanical stimuli, a process intrinsically linked to charge transfer. This perspective offers deeper insights into the charge-transfer mechanisms governing various ML phenomena, potentially expanding their applications in sensing, energy conversion, and environmental monitoring.

SL was first observed in water in 1934 by Frenzel and Schultes [15], who placed an ultrasound transducer in a tank of photographic developer fluid. While attempting to accelerate the development process, they noticed tiny dots on the film and realized that bubbles in the liquid emitted light when exposed to ultrasound. SL refers to the emission of intense bursts of light from ultrasonically driven collapsing gas bubbles in liquids, which can occur as either multi-bubble sonoluminescence (MBSL) or stable single-bubble sonoluminescence (SBSL) [16]. Due to the complex interactions among oscillating and collapsing bubbles, achieving an accurate mechanistic understanding of MBSL remains challenging. In contrast, SBSL, unaffected by neighboring bubbles, has been extensively studied, with proposed mechanisms including the hotspot hypothesis, bremsstrahlung radiation, and collision-induced radiation. One of the most widely accepted explanations for SL attributes light emission to electrical discharges [17]. It has been suggested that the emission arises from electrons confined within voids in the hot, dense fluid formed during the final stages of bubble collapse, where high-temperature ionization of bubble constituents generates free electrons. This model aligns with observed spectral distributions, power output, and emission timescales of single-cavitating rare gas bubbles [18]. Even though SL is currently limited by inefficiency, difficulty in control, and challenges in scaling up, the simplicity and low cost of SBSL systems offer significant potential for applications in luminescence lifetime measurements, providing compact, short-lived, and cost-effective light sources.

PL occurs when certain piezoelectric crystals, such as NaCl, KCl, KBr, and polycrystalline LiF (TLD-100) [18], undergo deformation, distortion, or fracture under strong mechanical forces [14,19,20]. Leider first reported the PL emission spectrum of KBr, observing a peak emission around 400 nm using a spectrograph [21]. Subsequent spectral studies by Butler on various alkali halides revealed similarities between PL spectra and afterglow luminescence, the latter persisting after irradiation ceases [22]. Senchukov and Shmurak [21] further demonstrated that the PL spectrum of Cu-doped KCl closely resembles its intracenter luminescence spectrum. Chandra et al. [23] observed that in irradiated KBr, KCl, KI, and NaCl crystals, PL occurs not only during stress application but also upon stress release [24,25]. In essence, PL, exhibited across diverse materials, originates from recombination processes involving electrons, holes, and impurity ion centers [26]. PL enables a rapid response to mechanical forces without requiring external excitation sources, such as light or electricity. This capability opens new opportunities for optoelectronic devices designed for real-time mechanical force detection [27]. However, challenges remain in enhancing its durability and energy-conversion efficiency.

Unlike PL, where a piezoelectric material emits light upon deformation, TL occurs when a material is mechanically fractured, torn, scratched, crushed, or rubbed [28]. This phenomenon is commonly observed when breaking sugar crystals or peeling adhesive tape. All forms of crystalline sugar, including rock candy, granulated sugar, and sugar wafers, emit light when broken or ground in a mortar. When sugar crystals are crushed, localized electrical fields separate positive and negative charges, which then generate sparks as they recombine. Particularly intriguing are cases where the emitted light is not only confined to the narrow fracture surfaces but also originates from within the bulk of the material [29]. Advances in TL research may enable the development of a new class of force-responsive sensors based on mechanical stimuli [30]. More recently, TIEL, which converts kinetic energy into light, has garnered significant attention. Its underlying mechanism is primarily attributed to the coupling effect between triboelectrification and electroluminescence. Triboelectrification, a charge-transfer process occurring when two materials come into contact or slide against each other, effectively converts mechanical energy into electrical energy, driving the subsequent luminescent response. Meanwhile, the transferred surface static charge can generate a strong local electric field within the material, accelerating high-energy electrons and inducing impact ionization, which excites luminescent centers and triggers electroluminescence. Trap-controlled materials [8] modulate luminescence kinetics through charge carrier trapping and release, while trap-type materials directly emit light from trap states themselves. Trap-type materials refer to a class of materials with special energy-level structures or defects that can capture and temporarily store charge carriers (such as electrons or holes), photon energy, or other excited particles. These materials regulate the absorption, storage, and release of energy through the “trap” mechanism and are widely used in semiconductor devices, optoelectronics, energy storage, and other fields. TIEL materials primarily consist of trap-type inorganic semiconductor electroluminescent materials doped with lanthanide luminescent elements and transition metal ions, such as ZnGa_2_O_4_:Mn^2+^, ZnAl_2_O_4_:Mn^2+^, ZnS:Cu/Mn, ZnS:Cu, and Al [31,32,33]. Driven by the alternating electric field generated during triboelectrification, these semiconductors exhibit high-field electroluminescence properties. Based on different mechanical triggering mechanisms, TIEL devices can be categorized into three modes: contact-sliding, contact-separation, and non-contact. These devices have broad applications in anti-counterfeiting, real-time vision sensing, human–machine interaction systems, and self-powered illumination and display technologies. Both TL and TIEL are promising phenomena that harness friction mechanical energy to produce light. While TL is simpler and does not rely on electrical-charge buildup, it suffers from low intensity and limited controllability. In contrast, TIEL holds promise for self-powered, low-energy systems with applications in sensing and energy harvesting, though its efficiency and material durability require improvement for broader adoption. Both technologies remain in the research phase, necessitating innovations in material science and system design to overcome their current limitations.

A straightforward and controllable luminescent reaction has been achieved through contact-electro-luminescence (CEL), which relies on interfacial electron transfer in solid–liquid CE rather than mechanical deformation or fracturing of materials. Triggered by electrostatic charge generated from CE between inert solid dielectrics and deionized (DI) water, reactive oxygen species (ROS) are produced, driving the CEL process. With the advantages of being metal-free and cost-effective, solid dielectrics offer a promising alternative to expensive metal-based catalysts, aligning with the principles of green chemistry and sustainable development. Notably, even the triboelectric charge generated from flowing luminol solutions within solid dielectric tubes can directly induce luminol luminescence [34]. Furthermore, the production of ROS can be modulated by varying solid dielectrics and additives, allowing precise control over the luminol reaction. CEL represents a promising and sustainable luminescence mechanism, enabling metal-free catalysis in green chemistry. However, its limited mechanistic understanding must be addressed to facilitate broader applications.

In summary, ML phenomena typically involve charge movement induced by mechanical stimuli, leading to light emission through mechanisms such as strong interfacial electric fields exciting surrounding gases, charge recombination, or the generation of ROS via electron transfer to luminescent oxides. Due to its advantages, including a wide selection of materials, low mechanical stimulus requirements, and flexible tunability, CEL has garnered significant attention in the ML field. Moreover, the chemical processes underlying CEL provide a novel platform for exploring the interfacial charge-transfer mechanisms of CE from a physicochemical perspective.

## 2. Chemical Reaction Initiated by Triboelectric Charge from CE

### 2.1. Contact-Electro-Chemistry (CE-Chemistry)

The phenomenon of CE, characterized by the generation of triboelectric charge at the interface between contacting materials, has been observed for over 2600 years since its initial discovery [12,35,36]. However, its crucial role in interfacial chemical reactions has often been overlooked. Only recently has the CE effect been recognized for its ability to drive interfacial chemistry, now termed “contact-electro-chemistry” (CE-Chemistry) [8,35,37]. When two materials interact under external mechanical force, their electron clouds overlap, lowering the energy barrier for charge transfer. If the input energy exceeds this barrier, electrons transfer from one atom to another, where they remain, enabling the generation of reactive oxygen species (ROS) such as hydroxyl radicals (^−^OH), superoxide radicals (^−^O_2_^−^), and singlet oxygen (^1^O_2_) to catalyze chemical reactions. CE-Chemistry has been reported to occur ubiquitously in both aqueous and non-aqueous systems to initiate various types of radical generation, facilitating redox reactions, organic polymerizations, and pollutant degradation, among others. It is thus not merely a physical process but also a complex physicochemical phenomenon involving charge transfer, radical generation, and subsequent chemical transformations. Distinct from conventional chemical reactions triggered by light, heat, electricity, or metal catalysis, CE-Chemistry, based on mechanical contact separation, offers a novel pathway for exploring interfacial interactions at the intersection of physics, materials science, and chemistry.

### 2.2. CEL

**Figure 2:**
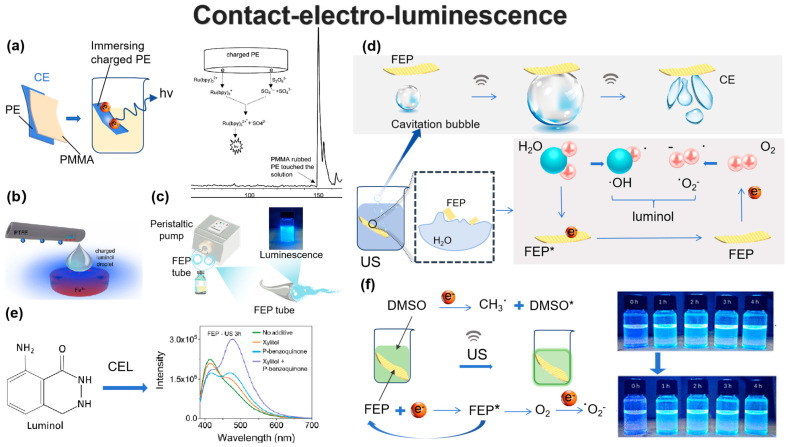
CEL initiated by triboelectric charge from CE. (**a**) Chemiluminescence induced by a PMMA-rubbed PE rod [38]. (**b**) Electrostatic charges regulate chemiluminescence by electron transfer at the liquid–solid interface [39]. (**c**) CEL induced by flow electrification in dielectric tubes [34]. (**d**) Schematic representing the CEL phenomenon during US. (**e**) CEL modulated by triboelectric charge [14]. (**f**) The improved CEL in non-aqueous systems [41].

The CEL strategy utilizes metal-free, cost-effective solid dielectrics as a sustainable alternative to expensive metal-based catalysts, aligning with the principles of green chemistry and sustainable development. Early studies by Liu et al. [38] demonstrated that electrostatic charges on dielectric surfaces, such as polymers like Teflon (polytetrafluoroethylene, PTFE) and Lucite (poly(methyl methacrylate), PMMA), could drive various chemical redox reactions, including metal deposition, ion reduction, and chemiluminescence. As shown in Figure 2a, when a polyethylene (PE) rod was first rubbed with PMMA, it became negatively charged due to the CE effect. Upon immersion in a solution containing 0.25 mM Ru(bpy)_3_(ClO_4_)_2_ and 2.5 mM Na_2_S_2_O_8_, a chemiluminescent emission was observed at the μA level, demonstrating the potential of CE-induced electrochemical reactions. In 2022, Zhang et al. [39] leveraged triboelectric charge transfer at the solid–liquid interface to investigate the role of intrinsic triboelectric charges within the solution itself. Their findings revealed that increased reaction activity and enhanced chemiluminescence occurred only when the luminol droplet carried a positive charge (Figure 2b), whereas negatively charged luminol, in contrast, inhibited chemiluminescence. This provided direct evidence that electrons serve as the charge carriers of triboelectricity at the liquid–solid interface. Their work introduced a novel strategy for electrostatically regulating chemiluminescence by simply charging the reaction solution with a dielectric solid. Furthermore, Xu et al. [34] developed a tunable and easily recyclable CEL method by utilizing triboelectric charges generated through flow electrification between moving liquids and solid dielectric tubes (Figure 2c). Fluorinated ethylene propylene (FEP) tubes functioned both as CE dielectrics and reaction vessels, allowing precise regulation of CE-Chemistry through physical parameters such as input flow velocity, tube inner diameter, length, and series-parallel configurations.

Ultrasonication is a widely employed mechanical method to enhance luminescence reactions in CEL. The propagation of ultrasonic waves in solution generates cavitation bubbles (CBs), whose collapse is hypothesized to induce frequent CE at the FEP–water interface, facilitating electron exchange [40]. As illustrated in Figure 2d, the collapse of CBs produces a high-pressure microjet that displaces previously adsorbed water molecules from the FEP surface. Upon contact, electron transfer occurs from water to FEP, leading to the formation of a charged state, denoted as FEP*. Concurrently, dissolved O_2_ is released and captures an electron from FEP* upon collision, restoring the FEP surface to its initial uncharged state. This process generates ^−^OH and ^−^O_2^−^_ radicals, which directly oxidize luminol to produce luminescence. In addition, the formation of ROS in this reaction can be modulated by various solid dielectrics and additives, enabling precise control over luminol luminescence. To better understand these intermediates and enhance the accuracy and reliability of luminol-based detection methods, Xu et al. [14] introduced additives such as xylitol and p-benzoquinone to directly monitor the luminol luminescence reaction. Two distinct luminescent emission peaks were observed, with their intensities tunable through the regulation of intermediate states (Figure 2e). The identification of these intermediates provides valuable insights into the fundamental mechanisms of luminescence reactions, facilitating the development of more effective and adaptable luminescence systems.

The CEL reaction solvent is typically limited to aqueous solutions, which readily form electrical double layers (EDLs) that hinder solid–liquid interfacial electron transfer and reduce reaction efficiency. Given that non-aqueous solvents play a critical role in chemical reactions—affecting reaction rates, product yields, and even mechanisms—Liu et al. [41] pioneered a CE-induced luminescent reaction in a non-aqueous system (Figure 2f). During the contact between FEP and dimethyl sulfoxide (DMSO), DMSO loses electrons, generating methyl radicals (CH_3^−^_) and other reactive DMSO derivatives (DMSO*), while FEP gains electrons, forming FEP*. Simultaneously, O_2_ captures electrons from FEP*, leading to the formation of ^−^O_2^−^_ radicals and restoring FEP* to its uncharged state. The non-aqueous solvent DMSO has been reported to effectively mitigate the EDL screening effect [8] and has been widely utilized as an organic medium for chemical reactions [31]. Utilizing non-aqueous solvents to circumvent the EDL screening effect presents a crucial strategy for enhancing reaction efficiency. The high and stable luminescence characteristics were maintained for 3 months, which improved the imaging accuracy and stability exponentially. This study expands the frontiers of CEL, providing an effective approach for tuning luminescence reaction selectivity and advancing our understanding of solvent effects in CEL mechanisms. CEL highlights the crucial role of triboelectric charge at the interface between inert dielectric and liquid, eliminating the need for chemical catalysts. Its advantages lie in the tunability of properties enabled by a wide selection of materials. As such, CEL establishes a new paradigm for luminescence and can be integrated with other chemical methods, such as electrochemistry and surface chemistry, to further explore its underlying mechanisms.

## 3. Conclusions

In summary, ML as a unique form of light emission induced by mechanical stimuli originates from complex interfacial charge-transfer and recombination processes. CEL, a subclass of ML, highlights the crucial role of CE in generating excitonic states, shedding new light on the interplay between mechanical deformation, charge accumulation, and radiative decay. Despite significant advances in understanding ML and CEL mechanisms, the intricate relationship between interfacial electron dynamics and luminescence efficiency remains elusive. Future research should focus on unraveling the quantitative correlation between charge-transfer efficiency and emission intensity, engineering materials with enhanced charge-separation characteristics, and exploring potential applications in stress sensing, optoelectronics, etc. Establishing a unified theoretical framework to describe interfacial charge transfer in ML systems will be key to unlocking their full potential in next-generation luminescent technologies.
